# Heterogeneity of *Campylobacter* species isolated from serial stool specimens of Egyptian children using pulsed field gel electrophoresis

**DOI:** 10.4102/ajlm.v2i1.34

**Published:** 2013-07-26

**Authors:** Atef M. El-Gendy, Momtaz O. Wasfy, Adel M. Mansour, Buhari Oyofo, Marwa M. Yousry, John D. Klena

**Affiliations:** 1 United States Naval Medical Research, Egypt; 2 Central Public Health Laboratories, Egypt; 3 United States Centers for Disease Control and Prevention, China

## Abstract

**Background:**

The genus *Campylobacter* spp. is a common cause of human acute bacterial enteritis and travellers’ diarrhoea worldwide.

**Objective:**

To determine whether multiple serial isolations of *Campylobacter* spp., when obtained from a single child, represented the same or a different organism.

**Methods:**

In a birth cohort study conducted in Egypt, numerous children showed serial isolations of *Campylobacter* spp. Of these, 13 children were selected from different households based on the successive isolation of six or more *Campylobacter* isolates from stool samples.

**Results:**

Eighty isolates were recovered and identified as either *Campylobacter coli* (*n* = 25) or *Campylobacter jejuni* (*n* = 55). Pulsed-field gel electrophoresis (PFGE) revealed the presence of 38 unique *C. jejuni* and 24 *C. coli* profiles at a similarity level of ≥ 90%. Although no serially-identical isolates were detected in six children, others demonstrated at least one identical couple of isolates; all identified serially between one to six weeks. Two children demonstrated > 80% similar couples of isolates that appeared seven months apart. PFGE could be a useful tool for differentiating reinfection, relapse and convalescent excretion phases.

**Conclusion:**

Our data suggest that *Campylobacter* infection in children is a complex process; children are exposed to multiple species in endemic environments and strains of the same bacterium appear to be shed serially between one to six weeks after the first exposure. Isolates that persisted for longer periods were relatively less similar, as shown from the results of this study.

## Introduction

The genus *Campylobacter* comprises a group of closely-related gram-negative bacteria which primarily colonise the gastrointestinal tracts of a wide variety of host species.^1^ *Campylobacter jejuni* and *Campylobacter coli* are common causes of human acute bacterial enteritis worldwide.^2^ In Egypt, the disease causes severe problems, with both acute and chronic clinical manifestations in infants aged less than two years, and is a known cause of travellers’ diarrhoea.^3,4^ Investigations have been complicated by the growth requirements of the bacteria, differences in pathogenic mechanisms and the possibility for molecular variability, constituting a substantial challenge for many scientific disciplines.^5^ Pulsed-field gel electrophoresis (PFGE) has been used for the molecular subtyping of *Campylobacter* species and is capable of intraspecies differentiation.^6^ In developed countries, most *Campylobacter* infections result from a unique exposure; reports of repeated infections are uncommon. The purpose of this study was to assess whether multiple serial isolations of *Campylobacter* spp., when obtained from a single child, represented the same or a different organism. We used PFGE analysis on a unique set of *Campylobacter* spp. isolates collected during a birth cohort study performed in the Nile Delta region of Egypt to answer this question. Participating children had diarrhoeal and non-diarrhoeal stool samples collected over a two-year period and the microbiological content of these samples was investigated. We selected a set of children who had a minimum of six successive *Campylobacter* spp. isolations during this time, and we examined these isolates by PFGE to determine if the isolates were the same species and the same pulsetype, based on SmaI restriction enzyme digestion.^7^

## Research method and design

### *Campylobacter* isolates

Isolates used in this study were collected from children participating in a birth cohort study conducted in a rural district of the Nile Delta, Egypt, between January 2004 and April 2007. Overall, 13 children showing six or more *Campylobacter* isolates with at least one diarrhoeal episode over a period of two years were chosen randomly for this study. The children resided in five different villages and had a median age of eight months (range 3–14 months) at first isolation of *Campylobacter* spp., and none were bottle-fed. Stool samples were generally collected every other week if no diarrhoea was reported. When diarrhoea was reported, two rectal swabs and a stool specimen were obtained.

*Campylobacter* colonies were recovered from Skirrow’s medium and confirmed by colonial morphology and gram stain, as well as oxidase and catalase activity.^8,9^ Speciation was performed using hippurate hydrolysis (positive for *C. jejuni* and negative for *C. coli* isolates)^8,10^ and multiplex polymerase chain reaction (mPCR) based on the nucleotide sequence of the lipid A gene, *lpxA*.^11^

### Antibiotic susceptibility

Antibiotic susceptibility measurements were made using the Epsilometer test (Etest) (AB Biodisk, Solna, Sweden) according to the manufacturer’s instructions. Etests were used to determine the minimum inhibition concentrations (MIC) of isolates against ciprofloxacin and erythromycin. Suspensions of the organisms were prepared in 5 ml of Mueller-Hinton broth and adjusted to a turbidity of 1 McFarland standard.^12,13^ The antibiotic susceptibility tests were performed from the adjusted inocula.^9^ Standardised bacterial suspensions were used to inoculate Mueller-Hinton agar supplemented with 5% defibrinated sheep blood. Excess moisture was allowed to be absorbed for about 10 to 15 minutes. When the surface of each plate had dried, Etest strips were placed aseptically on the plates (using sterile forceps). The plates were incubated within 15 minutes of applying the Etest strips in microaerophilic conditions (5% O_2_, 7.5% CO_2_, 7.5% H_2_, 80% N_2_) by using CampyPak Microaerophilic System Envelopes (Becton, Dickinson and Company, Loveton Circle Sparks, MD, USA) at 37 °C for 48 hours. MICs were read directly from the Etest strip at the point where the elliptical zone of inhibition intersects the MIC scale on the strip. Quality control strains (*C. jejuni* ATCC 33291 and *C. coli* ATCC 33559) were tested exactly the same as the clinical isolates^14^. MICs and disk-diffusion breakpoints for antibiotic resistance and susceptibility of *Campylobacter* isolates were determined according to Table 1 as described previously.^14^

**TABLE 1:** Breakpoints for antibiotic susceptibility and resistance.

Antibiotic	Disk diffusion Breakpoints for susceptibility	MICs		
		S	I	R
Erythromycin	≥ 20 mm	MICs of 0.06 to 4 µg/ml	-	MICs of ≥ 256 µg/ml
Ciprofloxacin	≥ 21 mm Intermediate = 17–21 mm	MICs of 0.06 to 0.25 µg/ml	MICs of 2 µg/ml	-

*Abbreviations*: R: Resistant, S: susceptible, I: intermediate.

### Pulsed-field gel electrophoresis

Intact deoxyribonucleic acid (DNA) was prepared from isolates from each child and digested as described previously^7^ using the U.S. Centers for Disease Control and Prevention (CDC) Pulsenet protocol for foodborne disease surveillance^7^ using a CHEF Mapper (Bio-Rad, CA, USA). Agarose gels were stained with ethidium bromide (50 µg/ml) and DNA band patterns were viewed by ultraviolet (UV) illumination. Gel images were captured and analysed using the BioNumerics Software package (version 5.10; Applied Maths, Austin, TX, USA). Similarity between pulsetypes was calculated using the Dice coefficient with a 2% tolerance for the band migration distance and clustering was performed using the complete linkage method (furthest neighbour) that considers the distance between any two given clusters as being the maximum distance between these clusters to reveal straggly clusters or outliers. This analysis method is considered to be a more strict interpretation of banding patterns than the unweighted pair group method using arithmetic averages (UPGMA).^7^

## Results

We examined 87 *Campylobacter* single-colony isolates collected from 13 children; 12 children showing six separate *Campylobacter* isolations, whilst 15 *Campylobacter* isolates were recovered from the remaining child over a period of 13 months. Of these isolates, seven were mixed *C. jejuni* and *C. coli* and were eliminated from further analysis.

**FIGURE 1:**
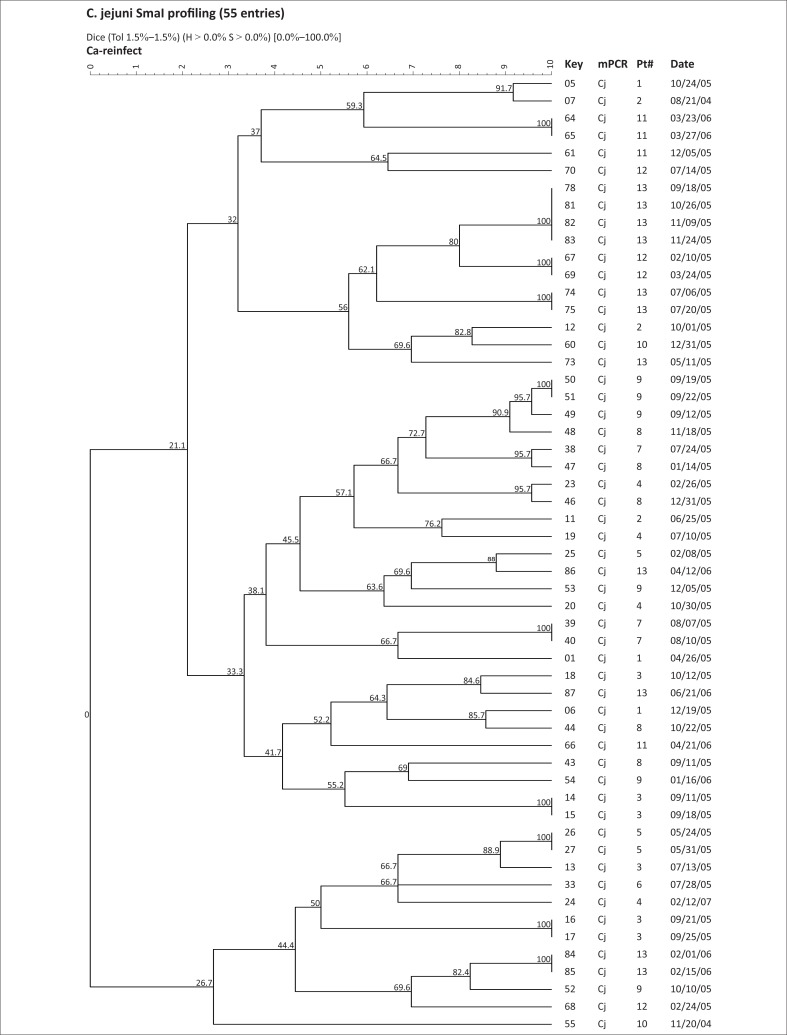
SmaI macrorestriction profiling of *C. jejuni* isolates by pulsed-field gel electrophoresis. Key, strain number; mPCR, multiplex PCR; Pt#, child number; Date, date of isolation at the US Naval Medical Research Unit No. 3, laboratory; CJ, *C. jejuni* Resistant.

**FIGURE 2:**
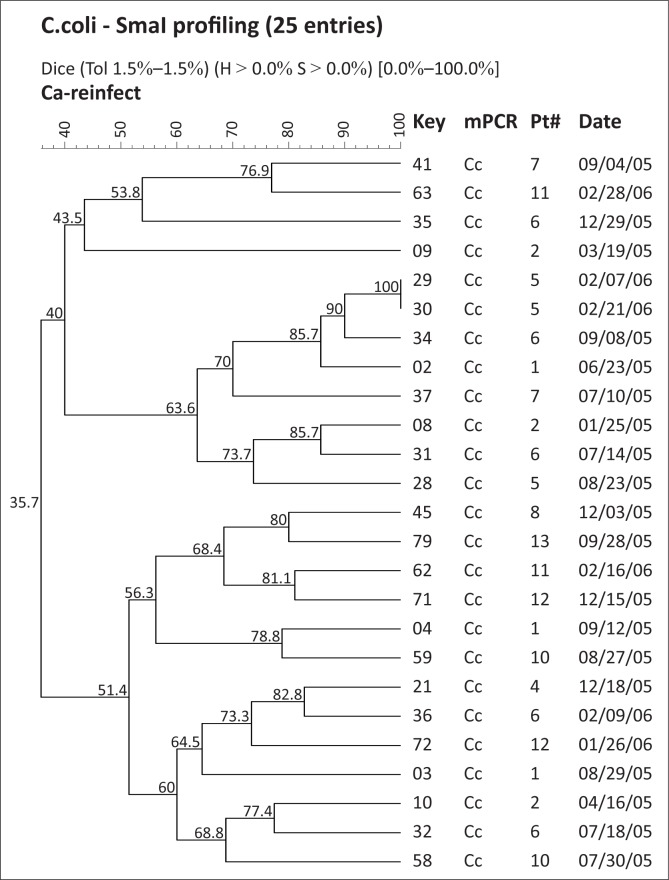
SmaI macrorestriction profiling of *C. coli* isolates by pulsed-field gel electrophoresis. Key, strain number; mPCR, multiplex PCR; Pt#, child number; Date, date of isolation at the US Naval Medical Research Unit No. 3, laboratory; CJ, *C. jejuni* Resistant.

**FIGURE 3:**
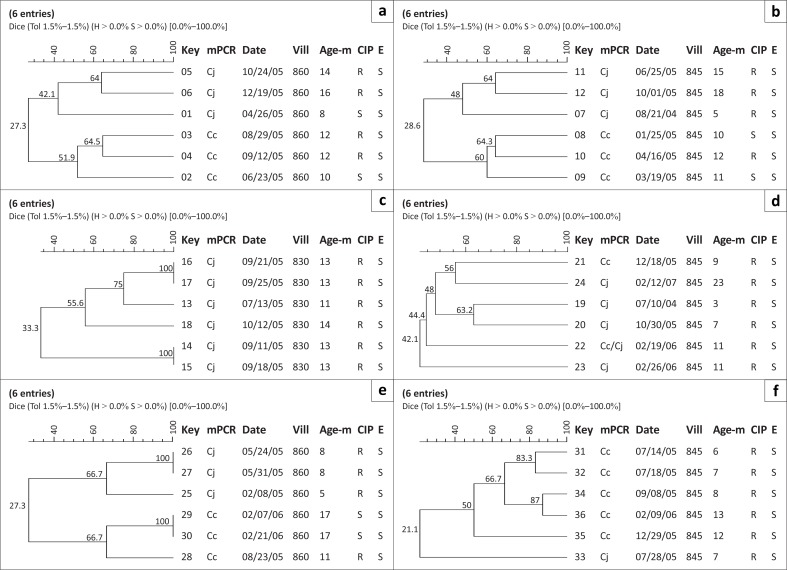
SmaI macrorestriction profiling of *Campylobacter* spp. isolates from (a) child 1, (b) child 2, (c) child 3, (d) child 4, (e) child 5, (f) child 6. *Abbreviations*: Key: strain #; mPCR: Date: date of isolation at the US Naval Medical Research Unit No. 3, laboratory; multiplex PCR, Pt#: child #, Vill: Village #, Age-m: Age in months, Cc: *C. coli*, CJ: *C. jejuni*, R: resistant, S: susceptible, CIP: ciprofloxacin, E: erythromycin.

**FIGURE 4:**
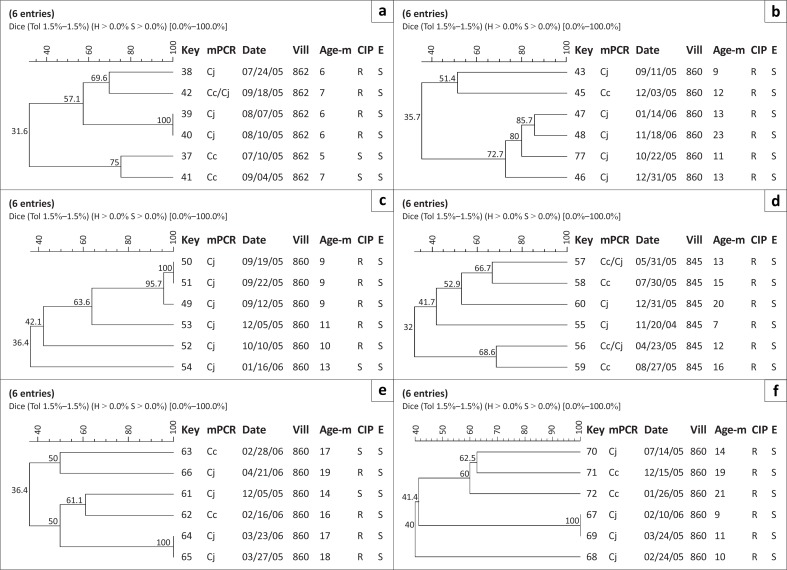
SmaI macrorestriction profiling of *Campylobacter* spp. isolates from (a) child 7, (b) child 8, (c) child 9, (d) child 10, (e) child 11, (f) child 12. *Abbreviations*: Key: strain #; mPCR: Date: date of isolation at the US Naval Medical Research Unit No. 3, laboratory; multiplex PCR, Pt#: child #, Vill: Village #, Age-m: Age in months, Cc: *C. coli*, CJ: *C. jejuni*, R: resistant, S: susceptible, CIP: ciprofloxacin, E: erythromycin.

**FIGURE 5:**
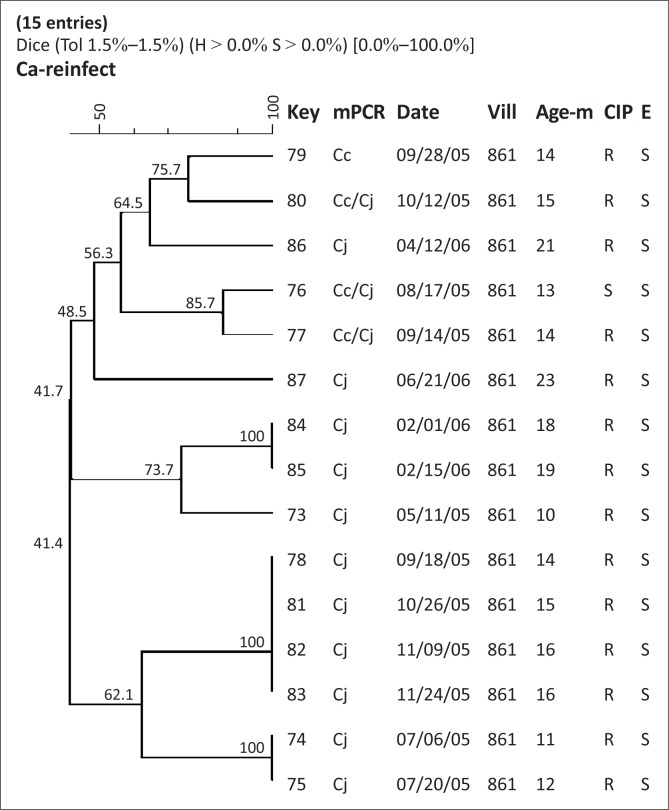
SmaI macrorestriction profiling of *Campylobacter* spp. isolates from Child 13. *Abbreviations*: Key: strain #; mPCR: multiplex PCR, Date: date of isolation at the US Naval Medical Research Unit No. 3, laboratory; Pt#: child #, Vill: Village #, Age-m: Age in months, Cc: *C. coli*, CJ: *C. jejuni*, R: resistant, S: susceptible, CIP: ciprofloxacin, E: erythromycin.

Amongst the 13 children, nine demonstrated diarrhoea due to *C. jejuni* (Child 1, 2, 3, 4, 5, 7, 8, 9, 13), three due to *C. coli* only (Child 6, 10, 12), and one child had episodes resulting from infection with both *Campylobacter* species (Child 11) (Figures 1–5). The number of diarrhoeal episodes varied amongst the children, with six showing a single episode (Child 1, 2, 5, 6, 8, 12) and three showing two episodes (Child 4, 7, 10), whilst two had three episodes (Child 9, 13) or even four (Child 3, 11).

Although no serially-identical isolates were detected in six children (Child 1, 2, 4, 6, 8, 10), others demonstrated at least one identical couple of isolates (Child 3, 5, 7, 9, 11, 12, 13), all identified at one to six week intervals from each other. Two children demonstrated ≥ 80% similar couples of isolates (Child 6, 8) that appeared seven months and one year apart, respectively. Child 7, 9, 11 and 12 had only five unique isolates, since each had one identical serial isolate that was recovered after one to six weeks. Only three children had more than one pair of indistinguishable *Campylobacter* isolates associated with their stools (Child 3, 5, 13). Confirmation of *Campylobacter* spp. isolates was conducted with the *lpxA* mPCR; 55 *C. jejuni* and 25 *C. coli* species were identified. Comparing phenotypic and genetic methods, seven *C. jejuni* isolates (8%) failed to hydrolyse hippurate and would have been misclassified using phenotypic methods alone. Most diarrhoeal episodes were due to *C. jejuni* (19/24, 79%); all six *Campylobacter* isolates recovered from two children (Child 3, 9) were *C. jejuni* and at least one *C. jejuni* isolate was recovered from the remaining 11 children, who also had at least one *C. coli* isolate recovered, although no child was exclusively positive for only *C. coli*.

Excluding the seven mixed infections, 86% (69/80) of the *Campylobacter* isolates were resistant to ciprofloxacin (CIP), an antibiotic often selected for treatment of campylobacteriosis.^15^ Only 5% (*n* = 3/55) of the *C. jejuni* isolates were susceptible to ciprofloxacin; however 32% (*n* = 8/25) of *C. coli* were susceptible. All isolates were susceptible to erythromycin. Table 2 shows the results of the susceptibility testing.

**TABLE 2:** Susceptibility testing of *Campylobacter* isolates.

Reaction	Disk Diffusion Test		MIC by Etest	
	Erythromycin	Ciprofloxacin	Erythromycin	Ciprofloxacin
Resistant	0	75 (86%)	0	75 (86%)
Susceptible	87 (100%)	12 (14%)	87 (100%)	12 (14%)

Because stool samples were collected from children routinely over a two-year period, we were able to look at whether a single *Campylobacter* isolate persisted in a child, or if that child was colonised (infected) by multiple isolates.

We utilised the discriminatory power of PFGE to assess the relationship of isolates recovered from each child (Figures 1–5), as well as to provide an overall picture of the *Campylobacter* population associated with the children (Figures 1 and 2). We grouped the children into four categories, namely, (1) children exposed to multiple species and strains of *Campylobacter* spp., (2) pre-diarrhoeal to diarrhoeal, (3) diarrhoeal-shedding and (4) active infection only.

### Exposure determined by pulsed-field gel electrophoresis analysis

Using PFGE at ≥ 90% similarity level, it was found that six children had six unique (dissimilar) *Campylobacter* isolates, (for example, the six isolates recovered from Child 1) (Isolates 1–6; Figure 3) were unique. The first isolate was recovered when Child 1 was eight months old and the last at 16 months. Child 1 was colonised by both *C. jejuni* (*n* = 3) and *C. coli* (*n* = 3) during this eight-month period and all of the isolates had distinguishable SmaI PFGE profiles. Only Isolate 6 (*C. jejuni*) was associated with diarrhoea. The same pattern was observed in Child 2, 4 and 8. Child 6 and 10 showed the same pattern, but demonstrated diarrhoea due to *C. coli*. The data suggest that children are exposed to a wide range of strains of *C. coli* and *C. jejuni*.

In Child 2, diarrhoea was associated with only one *C. jejuni* isolate (Isolate 12) that was CIP-resistant and 64% similar to a forerunner (Isolate 11), but appeared three months earlier. All *C. coli* isolates were recovered from that child without symptoms at one to three month intervals and exhibited 28% – 64% similarity. In Child 6 (Figure 3), diarrhoea was associated with a single *C. coli* isolate (Isolate 32) that was 83.3% similar to another (Isolate 31) that had been identified four days earlier and was 21.1% similar to a third (Isolate 33) recovered 10 days later. Child 8 (Figure 4) demonstrated one diarrhoeal episode due to *C. jejuni* (Isolate 44), whilst disease in Child 10 was associated with one *C. coli* isolate (Isolate 58) that was 32% similar to another *C. coli* successor (Isolate 59) isolated 28 days later.

### Recovery of pre-diarrhoeal to diarrhoeal isolates

Of the six isolates recovered from Child 7 (Figure 4), two (Isolate 38, 40) were associated with diarrhoea. Both isolates were *C. jejuni* and were recovered just over two weeks apart (24 July 2005 and 10 August 2005); however, these isolates were readily distinguishable by PFGE analysis (Figure 4). A third *C. jejuni* isolate (Isolate 39), recovered on 7 August 2005 from a routine (non-diarrhoeal) sample, was indistinguishable from Isolate 40. The scenario suggested by the PFGE patterns is that after infection by Isolate 38, Child 7 was re-colonised by a new strain of *Campylobacter* (Isolate 39) that was also able to cause an infection that resulted in diarrhoea (recovery of Isolate 40). Prior to and after these *C. jejuni* infections, *C. coli* was recovered from Child 7 (Isolate 37, 10 July 2005 and Isolate 41, 4 September 2005, respectively). Interestingly, the final isolate from Child 7 was a mixed infection (Isolate 42, 18 September 2005). It is unclear from the PFGE profile whether this mixed infection was two previously-documented isolates, new isolates, or a combination of old and new isolates. PFGE results from Child 7 (Figure 4) indicate a complicated microbiological milieu, since six *Campylobacter* isolates were recovered from this child over a three-month span. The first, Isolate 37 (*C. coli*), was not associated with diarrhoea, but Isolate 38 (*C. jejuni*), recovered 14 days later, was.

### Recovery of diarrhoea to shedding isolates

For Child 5, three *C. jejuni* and three *C. coli* isolates were recovered over a 12-month period (Figure 3). After initial recovery of a *C. jejuni* isolate (Isolate 25) that was not associated with diarrhoea, a second distinguishable *C. jejuni* isolate (Isolate 26), associated with diarrhoea, was recovered three months later. Isolate 26 had an indistinguishable pulsetype from Isolate 27, a non-diarrhoea-associated *C. jejuni* recovered one week later. One distinguishable *C. jejuni* and three distinct *C. coli* isolates were recovered prior to and after the *Campylobacter*-associated diarrhoea caused by Isolate 26. In addition, the recovery of indistinguishable Isolates 29 and 30 two weeks apart (Figure 3) highlights the possibility of carriage of *Campylobacter* spp. for extended periods of time, although not necessarily associated with diarrhoea.

A similar story is presented by Child 13, from whom 15 *Campylobacter* isolates were recovered. This child demonstrated 11 serial isolates of *C. jejuni* (Figure 5). Of these, two identical strains (Isolate 74, 75) were recovered at an interval of two weeks, but did not cause diarrhoea. Two months later, two diarrhoeal episodes occurred as a result of infection by an identical couple of isolates (Isolate 78, 81) that persisted for four weeks without diarrhoea (Isolate 82, 83). Three months later, two identical strains (Isolate 84, 85) persisted for two weeks with 41.4% similarity to the preceding two clusters (Isolate 74, 75, 78, 81, 82, 83).

Child 13 continued to shed Isolate 81 for nearly a month (along with recovery of indistinguishable Isolates 82 and 83 up until 24 November 2005), after which time we no longer recovered this pulsetype.

The data from these two children suggest that *C. jejuni* can be shed for at least one week and for as long as six weeks after active infection. In a *Campylobacter*-endemic environment, it also appears that multiple colonisation events, involving different species and strains, can take place during this time.

Child 12 continued to shed *C. jejuni* Isolate 67 for nearly six weeks (recovery of indistinguishable isolates 67 and 69, 24 March 2005); however, this child only demonstrated diarrhoea resulting from *C. coli* infection (Isolate 71), recovered 9 months later.

### Only active diarrhoea observed

Child 3 (Figure 3) had five *C. jejuni* isolates associated with diarrhoea (Isolate 14, 15, 16, 17, 18), with two pairs (Isolates 14 and 15, 16 and 17) being indistinguishable from each other by PFGE analysis. In each case, the respective pairs were recovered less than a week apart (11 September 2005 and 18 September 2005; 21 September 2005 and 25 September 2005). *C. jejuni* was also recovered prior to the first diarrhoeal episode (Isolate 13, 13 July 2005), but this isolate was distinguishable from all of the other recovered isolates from Child 3. Additionally, Isolate 18, recovered after Isolate 17 (12 October 2005), was also associated with diarrhoea. Isolate 18 was also distinct from all other Child 3 isolates.

Likewise, the remaining two children (Child 9, 11) had multiple isolates associated with diarrhoea and whilst we were able to recover *Campylobacter* spp. after these diarrhoeal episodes, we could not recover an isolate with a pulsetype that was distinguishable from the diarrhoeal cause.

In Child 11, a single diarrhoeal episode resulted in the recovery of two indistinguishable *C. jejuni* isolates (Isolate 64, 65; Figure 4). However, these isolates were distinct from two *Campylobacter*-associated diarrhoea isolates (Isolate 62, 63), both of which were *C. coli*. PFGE analysis also suggests that Isolates 62 and 63 were distinct; indicating that Child 11 had three consecutive *Campylobacter*-associated diarrhoeas involving two species and three unique isolates. Whilst a final *C. jejuni* was recovered from Child 11 after the diarrhoea caused by Isolates 64 and 65, this isolate had a unique PFGE pattern. Child 9 was unique in that only *C. jejuni* (*n* = 6) was recovered from this child who showed three successive episodes (Isolates 50–52; CIP-resistant).

## Ethical considerations

This study was supported by the U.S. Department of Defense (DoD) Global Emerging Infections System (GEIS), DoD 31934 NAMRU3. 2003. Ethical considerations and informed consent forms were reviewed and approved by both the Institutional Review Board (IRB) of the US Naval Medical Research Unit No. 3 and the Egyptian Ministry of Health in compliance with all Federal regulations governing the protection of human subjects (IRB Protocol No.145, step D, titled: ‘Natural Immunity to ETEC Infections in Egyptian Infants and Children’.

Voluntary participation was adopted, with freedom to withdraw. No major lapses were observed. Informed consent was obtained from parents or legal guardians of minors. No hazards were entailed in subject participation, but patients, physicians and the community could benefit from the results with regard to disease treatment, diagnosis and control.

Before sending samples to the laboratory, the privacy and confidentiality of the participating subjects were maintained by removing any direct personal identifiers and replacing them with codes (letters and numbers), not derived from or related to the personal information. The original identifiers were maintained securely and only traced back to the source at the physician’s discretion for treatment or control purposes.

## Discussion

Worldwide, thermotolerant *Campylobacter* species are amongst the most common causative agents of gastroenteritis.^11,16^ The main sources of infection include the consumption of unpasteurised milk, undercooked poultry or beef and contaminated water.^17^ In a two-year cohort study conducted in Egypt, many children showed serial isolations of *Campylobacter* spp., with at least one diarrhoeal episode (range=1–4 episodes per child), which may reflect the varying degrees of pathogenicity amongst *Campylobacter* isolates as well as the limited role of cross-protective mechanisms. This investigation attempted to explore whether these isolates represented the same or different infections. Previous studies have demonstrated age-related immunity, with a decrease in the case-to-infection ratio, absence of symptoms and shorter convalescent-phase excretion.^18,19^ This may or may not be supported by the observation that the enrolled children had a median age of eight months (range 3 months – 14 months) at first isolation of *Campylobacter*, whilst the study had lasted for two years.

The species of all *Campylobacter* isolates were confirmed by the *lpxA* mPCR. We found that seven *C. jejuni* isolates detected by this method were negative using the hippurate hydrolysis test, which may be due to the variable expression levels of the N-benzoylglycine amidohydrolase (hippuricase) gene.^20^ This finding supports the use of a genetic-based approach for the rapid and accurate identification of thermotolerant *Campylobacter*.^8,21^

*C. jejuni* isolates were the most commonly-recovered campylobacters^22,23^ and were most frequently associated with diarrhoeal episodes (79%) amongst the 13 children. However, *C. coli* was responsible for diarrhoea in only three children, suggesting that although not as dominant as *C. jejuni*, the opportunity for environmental exposure to *C. coli* is significant in children in the Nile Delta. Globally, *C. jejuni* has been reported to cause more than 90% of *Campylobacter* infections,^22,24^ regardless of the severity of the clinical picture.^18^

To define the relationship amongst the *Campylobacter* isolates more accurately overall and from each individual child, isolates were pulsetyped using a protocol developed by PulseNet International that is specific for *Campylobacter* testing.^7^ The restriction enzyme, SmaI, was used since other options have been shown to provide less reproducible results.^25^ Analysing the profiles obtained per child, it appears that identical *C. jejuni* isolates (96% – 100%) were recovered frequently during diarrhoea episodes (as in Child 5, 9 and 12) for intervals ranging between one and six weeks, whilst *C. coli* isolates were identical in only one child (Child 5), within two weeks. However, most *C. coli* isolates were distinguishable, with 50% – 67% PFGE band similarity, when recovered at periods from two weeks to seven months.

Assuming that an episode of diarrhoea may last for an average of one week,^26^ isolation of an identical strain after longer periods without diarrhoea may be regarded as being a convalescent-phase excretion.^27,28,29^ For instance, in Child 13, indistinguishable *C. jejuni* isolates were recovered up to nine weeks after a diarrhoeal episode (Isolates 78 and 81–83). This is longer than reported earlier,^3^ which marked the presence of convalescent-phase excretion for only four weeks. However, at least one other study reported the shedding of *Campylobacter* isolates for more than nine months post-infection.^27^ In our study, Child 6 showed isolates with > 83% similarity that were identified seven months post-infection.

Reinfection with *C. jejuni* spp. was observed in Child 3, 4, 7 and 9, who developed diarrhoeal episodes in association with weakly-related isolates (< 33%, 42%, 31%, and 36% band-matching, respectively) that appeared after variable periods of shedding, ranging from three days to 18 months. Child 11 was similar, but four consecutive diarrhoeal episodes occurred due to both *C. jejuni* and *C. coli* isolates (36% similarity), with one to two week intervals between each. In Child 13, two identical *C. jejuni* CIP-resistant isolates were recovered from diarrhoeal episodes that took place nine weeks apart (September 2005 and 24 November), which may be regarded as recrudescence. This observation may be attributed to the lack of an adaptive immune response or resistance to antibiotic treatment.^30,31^ Prolonged *Campylobacter* diarrhoeal attacks have been reported to last for about 12 days only and were associated with CIP-resistant *Campylobacter*.^32^

Three children (Child 3, 7, 9) showed isolates identical to those causing diarrhoea, but at an earlier time before disease (10 days, three days and one week, respectively), which may be explained by the presence of a colonisation phase during the incubation period.^33^ It is also possible that pre-shedding of distinguishable *Campylobacter* strains is an *in vivo* predisposition through mutations at multiple sites (13 contingency loci) to augment bacterial virulence and speed up adaptation to the host.^34,35^ For instance, our PFGE results from Child 7 show that two indistinguishable *C. jejuni* isolates (Isolates 39 and 40) were distinct from an earlier isolate (Isolate 38), suggesting that Isolate 38 was rapidly replaced or dominated by the more invasive *C. jejuni* Isolate 39, which resulted eventually in a second diarrhoeal episode.

Overall, this study identified 80 *Campylobacter* spp. isolates from 13 children showing six serial isolations during a two-year study. The bacteria comprised 38 unique SmaI-PFGE profiles of *C. jejuni* and 24 profiles of *C. coli* at a similarity value of > 90% as described previously.^36^ This supports an immense genotypic diversity amongst *Campylobacter* species, an observation that has been attributed to the occurrence of inter- and intra-strain genetic exchanges during infection.^28^ Some children showed reinfection after variable periods from the first episode, whilst relapse was noted after seven weeks. Additional studies using multiple isolates from the same subject are required to advance our understanding of *Campylobacter* infection and immunity. Our data suggest that *Campylobacter* infection in children in endemic environments is a complex process; when children are exposed to multiple species and strains of *Campylobacter*, the same bacterium appears to be shed serially for between one to six weeks after the first exposure. Isolates that persisted for longer periods were relatively less similar as shown from the results of this study. Further detailed evaluation of the epidemiology and the isolates associated with diarrhoea and from non-diarrhoeal periods will be necessary in order to determine risk factors and mechanisms to prevent or control re-exposure of children in these settings.

## References

[1] Blaser MJ Epidemiologic and clinical features of Campylobacter jejuni infections. J Infect Dis. 1997;176 Suppl 2:S103–105. http://dx.doi.org/10.1086/513780, PMid:9396691 10.1086/513780

[2] Wasfy MO, Oyofo BA, David JC, et al Isolation and antibiotic susceptibility of Salmonella, Shigella, and Campylobacter from acute enteric infections in Egypt. J Health Popul Nutr. 2000;18(1):33–38. PMid:11014768

[3] Rao MR, Naficy AB, Savarino SJ, et al Pathogenicity and convalescent excretion of Campylobacter in rural Egyptian children. Am J Epidemiol. 2001;154(2):166–173. http://dx.doi.org/10.1093/aje/154.2.166, PMid:11447051 10.1093/aje/154.2.166

[4] Putnam SD, Frenck RW, Riddle MS, et al Antimicrobial susceptibility trends in Campylobacter jejuni and Campylobacter coli isolated from a rural Egyptian pediatric population with diarrhea. Diagn Microbiol Infect Dis. 2003;47(4):601–608. http://dx.doi.org/10.1016/S0732-8893(03)00154-8 14711482 10.1016/s0732-8893(03)00154-8

[5] Praakle-Amin K, Roasto M, Korkeala H, et al PFGE genotyping and antimicrobial susceptibility of Campylobacter in retail poultry meat in Estonia. Int J Food Microbiol. 2007;114(1):105–112. http://dx.doi.org/10.1016/j.ijfoodmicro.2006.10.034, PMid:17182145 10.1016/j.ijfoodmicro.2006.10.034

[6] Ono K, Kurazono T, Niwa H, et al Comparison of three methods for epidemiological typing of Campylobacter jejuni and C. coli. Curr Microbiol. 2003;47 (5):364–371. http://dx.doi.org/10.1007/s00284-002-4037-6, PMid:14669910 10.1007/s00284-002-4037-6

[7] Ribot EM, Fitzgerald C, Kubota K, et al Rapid pulsed-field gel electrophoresis protocol for subtyping of Campylobacter jejuni. J Clin Microbiol. 2001;39(5): 1889–1894. http://dx.doi.org/10.1128/JCM.39.5.1889-1894.2001, PMid:, PMCid:11326009 10.1128/JCM.39.5.1889-1894.2001 PMC88044

[8] Nakari UM, Puhakka A, Siitonen A Correct identification and discrimination between Campylobacter jejuni and C. coli by a standardized hippurate test and species-specific polymerase chain reaction. Eur J Clin Microbiol Infect Dis. 2008;27(7): 513–518. http://dx.doi.org/10.1007/s10096-008-0467-9, PMid:18317822 10.1007/s10096-008-0467-9

[9] Saenz Y, Zarazaga M, Lantero M, et al Antibiotic resistance in Campylobacter strains isolated from animals, foods, and humans in Spain in 1997–1998. Antimicrob Agents Chemother. 2000;44(2):267–271. http://dx.doi.org/10.1128/AAC.44.2.267-271.2000, PMid:, PMCid:10639348 10.1128/aac.44.2.267-271.2000 PMC89669

[10] Morris GK, el Sherbeeny MR, Patton CM, et al Comparison of four hippurate hydrolysis methods for identification of thermophilic Campylobacter spp. J Clin Microbiol. 1985;22(5):714–718. PMid:, PMCid:3902875 10.1128/jcm.22.5.714-718.1985 PMC268512

[11] Klena JD, Parker CT, Knibb K, et al Differentiation of Campylobacter coli, Campylobacter jejuni, Campylobacter lari, and Campylobacter upsaliensis by a multiplex PCR developed from the nucleotide sequence of the lipid A gene *lpxA*. J Clin Microbiol. 2004;42(12):5549–5557. http://dx.doi.org/10.1128/JCM.42.12.5549-5557.2004, PMid:, PMCid:15583280 10.1128/JCM.42.12.5549-5557.2004 PMC535264

[12] Ge B, Bodeis S, Walker RD, et al Comparison of the Etest and agar dilution for in vitro antimicrobial susceptibility testing of Campylobacter. J Antimicrob Chemother. 2002;50(4):487–494. http://dx.doi.org/10.1093/jac/dkf162, PMid:12356792 10.1093/jac/dkf162

[13] Luangtongkum T, Morishita TY, El-Tayeb AB, et al Comparison of antimicrobial susceptibility testing of Campylobacter spp. by the agar dilution and the agar disk diffusion methods. J Clin Microbiol. 2007;45(2):590–594. http://dx.doi.org/10.1128/JCM.00986-06, PMid:, PMCid:17122005 10.1128/JCM.00986-06 PMC1829028

[14] Gaudreau C, Girouard Y, Ringuette L, et al Comparison of disk diffusion and agar dilution methods for erythromycin and ciprofloxacin susceptibility testing of Campylobacter jejuni subsp. jejuni. Antimicrob Agents Chemother. 2007; 51(4):1524–1526. http://dx.doi.org/10.1128/AAC.00905-06, PMid:, PMCid:17261628 10.1128/AAC.00905-06 PMC1855498

[15] Lutgen EM, McEvoy JM, Sherwood JS, et al Antimicrobial resistance profiling and molecular subtyping of Campylobacter spp. from processed turkey. BMC Microbiol. 2009;9:203. http://dx.doi.org/10.1186/1471-2180-9-203, PMid:, PMCid:19772592 10.1186/1471-2180-9-203 PMC2758883

[16] Allos BM Campylobacter jejuni infections: update on emerging issues and trends. Clin Infect Dis. 2001;32(8):1201–1206. http://dx.doi.org/10.1086/319760, PMid:11283810 10.1086/319760

[17] Denis M, Chidaine B, Laisney MJ, et al Comparison of genetic profiles of Campylobacter strains isolated from poultry, pig and Campylobacter human infections in Brittany, France. Pathol Biol (Paris). 2009;57(1):23–29. http://dx.doi.org/10.1016/j.patbio.2008.04.007, PMid:18534783 10.1016/j.patbio.2008.04.007

[18] Carvalho AC, Ruiz-Palacios GM, Ramos-Cervantes P, et al Molecular characterization of invasive and noninvasive Campylobacter jejuni and Campylobacter coli isolates. J Clin Microbiol. 2001;39(4):1353–1359. http://dx.doi.org/10.1128/JCM.39.4.1353-1359.2001, PMid:, PMCid:11283056 10.1128/JCM.39.4.1353-1359.2001 PMC87939

[19] van Vliet AH, Ketley JM Pathogenesis of enteric Campylobacter infection. Symp Ser Soc Appl Microbiol. 2001;30:45S–56S. http://dx.doi.org/10.1046/j.1365-2672.2001.01353.x, PMid:11422560 10.1046/j.1365-2672.2001.01353.x

[20] Hani EK, Chan VL Expression and characterization of Campylobacter jejuni benzoylglycine amidohydrolase (hippuricase) gene in Escherichia coli. J Bacteriol. 1995;177(9):2396–2402. PMid:, PMCid:7730270 10.1128/jb.177.9.2396-2402.1995 PMC176897

[21] Willoughby K, Nettleton PF, Quirie M, et al A multiplex polymerase chain reaction to detect and differentiate Campylobacter fetus subspecies fetus and Campylobacter fetus -species venerealis: use on UK isolates of C. fetus and other Campylobacter spp. J Appl Microbiol. 2005;99(4):758–766. http://dx.doi.org/10.1111/j.1365-2672.2005.02680.x, PMid:16162226 10.1111/j.1365-2672.2005.02680.x

[22] Wardak S, Duda U, Krasowska D, et al [Campylobacter spp. as a leading cause of human bacterial gastroenteritis in selected region of Poland]. Przegl Epidemiol. 2009;63(4):531–537. PMid:20120952

[23] Wardak S, Szych J [Prevalence of pathogenic genes of Campylobacter jejuni isolated from humans in Poland between 2003–2005]. Med Dosw Mikrobiol. 2006;58(3):217–222. PMid:17340996

[24] Wardak S, Szych J, Sadkowska-Todys M The first report on Campylobacter coli family outbreak detected in Poland in 2006. Euro Surveill. 2008;13(9):pii:8052. PMid:18445405

[25] Clark CG, Price L, Ahmed R, et al Characterization of waterborne outbreak-associated Campylobacter jejuni, Walkerton, Ontario. Emerg Infect Dis. 2003; 9(10):1232–1241. http://dx.doi.org/10.3201/eid0910.020584, PMid:, PMCid:14609457 10.3201/eid0910.020584 PMC3033067

[26] Cawthraw SA, Feldman RA, Sayers AR, et al Long-term antibody responses following human infection with Campylobacter jejuni. Clin Exp Immunol. 2002;130(1): 101–106. http://dx.doi.org/10.1046/j.1365-2249.2002.01966.x, PMid:, PMCid:12296859 10.1046/j.1365-2249.2002.01966.x PMC1906500

[27] Albert MJ, Leach A, Asche V, et al Serotype distribution of Campylobacter jejuni and Campylobacter coli isolated from hospitalized patients with diarrhea in central Australia. J Clin Microbiol. 1992;30(1):207–210. PMid:, PMCid:1370848 10.1128/jcm.30.1.207-210.1992 PMC265021

[28] de Boer P, Wagenaar JA, Achterberg RP, et al Generation of Campylobacter jejuni genetic diversity in vivo. Mol Microbiol. 2002;44(2):351–359. http://dx.doi.org/10.1046/j.1365-2958.2002.02930.x, PMid:11972775 10.1046/j.1365-2958.2002.02930.x

[29] Yabe S, Higuchi W, Iwao Y, et al Molecular typing of Campylobacter jejuni and C. coli from chickens and patients with gastritis or Guillain-Barré syndrome based on multilocus sequence types and pulsed-field gel electrophoresis patterns. Microbiol Immunol. 2010;54(6):362–367. http://dx.doi.org/10.1111/j.1348-0421.2010.00222.x, PMid:20536735 10.1111/j.1348-0421.2010.00222.x

[30] Tribble DR, Sanders JW, Pang LW, et al Traveler’s diarrhea in Thailand: randomized, double-blind trial comparing single-dose and 3-day azithromycin-based regimens with a 3-day levofloxacin regimen. Clin Infect Dis. 2007;44(3):338–346. http://dx.doi.org/10.1086/510589, PMid:17205438 10.1086/510589

[31] Tribble DR, Baqar S, Carmolli MP, et al Campylobacter jejuni strain CG8421: a refined model for the study of Campylobacteriosis and evaluation of Campylobacter vaccines in human subjects. Clin Infect Dis. 2009;49(10):1512–1519. http://dx.doi.org/10.1086/644622, PMid:19842970 10.1086/644622

[32] Nelson JM, Smith KE, Vugia DJ, et al Prolonged diarrhea due to ciprofloxacin-resistant campylobacter infection. J Infect Dis. 2004;190(6):1150–1157. http://dx.doi.org/10.1086/423282, PMid:15319866 10.1086/423282

[33] Jones FR, Baqar S, Gozalo A, et al New World monkey Aotus nancymae as a model for Campylobacter jejuni infection and immunity. Infect Immun. 2006;74(1):790–793. PMid:, PMCid:16369042 10.1128/IAI.74.1.790-793.2006 PMC1346678

[34] Luo N, Sahin O, Lin J, et al In vivo selection of Campylobacter isolates with high levels of fluoroquinolone resistance associated with gyrA mutations and the function of the CmeABC efflux pump. Antimicrob Agents Chemother. 2003;47(1): 390–394. http://dx.doi.org/10.1128/AAC.47.1.390-394.2003, PMid:, PMCid:12499221 10.1128/AAC.47.1.390-394.2003 PMC148968

[35] Jerome JP, Bell JA, Plovanich-Jones AE, et al Standing genetic variation in contingency loci drives the rapid adaptation of Campylobacter jejuni to a novel host. PLoS ONE. 2011;6(1):e16399. http://dx.doi.org/10.1371/journal.pone.0016399, PMid:, PMCid:21283682 10.1371/journal.pone.0016399 PMC3025981

[36] Nadeau E, Messier S, Quessy S Comparison of Campylobacter isolates from poultry and humans: association between in vitro virulence properties, biotypes, and pulsed-field gel electrophoresis clusters. Appl Environ Microbiol. 2003;69(10): 6316–6320. http://dx.doi.org/10.1128/AEM.69.10.6316-6320.2003, PMid:, PMCid:14532099 10.1128/AEM.69.10.6316-6320.2003 PMC201179

